# Biochemical isolation and purification of ovulation-inducing factor (OIF) in seminal plasma of llamas

**DOI:** 10.1186/1477-7827-9-24

**Published:** 2011-02-10

**Authors:** Marcelo H Ratto, Louis TJ Delbaere, Yvonne A Leduc, Roger A Pierson, Gregg P Adams

**Affiliations:** 1 Faculty of Veterinary Sciences, Universidad Austral de Chile, Valdivia, Chile; 2 Department of Biochemistry, University of Saskatchewan, Saskatoon, Canada; 3 Department of Obstetrics Gynecology and Reproductive Science, University of Saskatchewan, Saskatoon, Canada; 4 Department of Veterinary Biomedical Sciences, University of Saskatchewan, Saskatoon, Canada

## Abstract

**Background:**

The objective of the present study was to isolate and purify the protein fraction(s) of llama seminal plasma responsible for the ovulation-inducing effect of the ejaculate.

**Methods:**

Semen collected from male llamas by artificial vagina was centrifuged and the seminal plasma was harvested and stored frozen. Seminal plasma was thawed and loaded onto a Type 1 macro-prep ceramic hydroxylapatite column and elution was carried out using a lineal gradient with 350 mM sodium phosphate. Three protein fractions were identified clearly (Fractions A, B, and C), where a prominent protein band with a mass of 14 kDa was identified in Fraction C. Fraction C was loaded into a sephacryl gel filtration column for further purification using fast protein liquid chromatography (FPLC). Isocratic elution resulted in 2 distinct protein fractions (Fractions C1 and C2). An in vivo bioassay (n = 10 to 11 llamas per group) was used to determine the ovarian effect of each fraction involving treatment with saline (negative control), whole seminal plasma (positive control), or seminal plasma Fractions A, B or C2. Ultrasonography was done to detect ovulation and CL formation, and blood samples were taken to measure plasma progesterone and LH concentrations.

**Results:**

Ovulation and CL formation was detected in 0/10, 10/11, 0/10, 2/11, and 10/11 llamas treated with saline, whole seminal plasma, Fractions A, B and C2 respectively (P < 0.001). A surge in circulating concentrations of LH was detected within 2 hours only in llamas treated with either whole seminal plasma or Fraction C2. Plasma progesterone concentration and CL diameter profiles were greatest (P < 0.05) in llamas treated with Fraction C2.

**Conclusion:**

Ovulation-inducing factor was isolated from llama seminal plasma as a 14 kDa protein molecule that elicits a preovulatory LH surge followed by ovulation and CL formation in llamas, suggesting an endocrine effect at the level of the hypothalamus (release of GnRH) or the pituitary (gonadotrophs).

## Background

Historically, the role of seminal plasma has been attributed primarily to its direct effects on spermatozoa; i.e., buffering, nutrition, capacitation, and transport [1]. However, recent findings about the systemic effects of seminal plasma in the female suggest an additional role - as an inducer of ovulation. The first direct evidence of an ovulation-inducing factor (OIF) in semen came from workers in China who reported that ovulation occurred after intravaginal or intramuscular administration of Bactrian seminal plasma to female Bactrian camels [2-4]. The existence of the putative OIF garnered little scientific attention for 20 years, until it was confirmed in a series of studies involving llamas and alpacas, New World relatives of camels [5]. Results from this and subsequent studies document that OIF 1) exists in semen of alpacas, llamas and koalas (induced ovulators), 2) is a potent stimulator of LH secretion, 3) has a dose-dependent effect on ovulation rate and CL form and function, and 4) acts via a systemic rather than a local pathway, at physiologically relevant doses [5-9].

The discovery of OIF in seminal plasma was made in species categorized as induced ovulators since factors influencing the occurrence of ovulation can be studied without the confounding effects of spontaneous ovulation. Results of recent studies, however, support the hypothesis that OIF in seminal plasma is conserved among species, including those considered to be spontaneous ovulators (e.g., bovine, equine and porcine; [8,10]). Furthermore, OIF in seminal plasma influenced ovarian function in species considered to be spontaneous ovulators; i.e., it induced ovulation in a pre-pubertal mouse model [11] and altered ovarian follicular wave dynamics in cows through a suppressive effect on the dominant follicle [12].

Attempts have been made to isolate and purify OIF in camel seminal plasma using a combination of anion exchange and hydrophobic chromatography [13,14]; however, interpretation of the results is limited because of the lack of a validated bioassay to quantitatively test the effects of various fractions. The initial supposition that OIF is related to the GnRH peptide is reasonable based on LH-releasing effects on pituitary cells and the presence of GnRH immuno-reactivity in human seminal plasma [15,16]. However, the addition of GnRH antibodies to in vitro rat pituitary cell culture did not block the LH-releasing effect of alpaca seminal plasma [17], suggesting that OIF has a different chemical structure than the classic peptide.

The biochemical identity of OIF in seminal plasma remains unknown. Using a systematic approach to ablate the bioactivity of llama seminal plasma, results of a recent study document that OIF is a protein molecule that is resistant to heat and enzymatic digestion with proteinase K, and has a molecular mass of more than about 30 kDa [18]. The objective of the present study was to isolate and purify the protein fraction(s) of llama seminal plasma responsible for the ovulation-inducing effect of the ejaculate.

## Methods

### Semen collection and preparation

Semen was collected from 3 male llamas (n = 24 ejaculates per male) over a period of 3 months at the University of Saskatchewan. Ejaculates were collected by artificial vagina, and the seminal plasma (total volume approximately 144 ml) was harvested after centrifugation, diluted, and stored frozen, as described previously [5]. Upon thawing, the seminal plasma was pooled and sonicated to reduce viscosity. Sonification was done on ice using a Sonifier Cell Disruptor with a microtip (Model W185, Heat Systems-Ultrasonics, Inc., Plainview, NY, USA) and applying five 15-second episodes at 70% of maximum power (35 watts) with 45-second rest periods between episodes. After sonification, the seminal plasma was centrifuged at 10,000 × g for 20 minutes to remove particulate matter.

### Hydroxylapatite column chromatography

Seminal plasma was loaded onto a Type 1 macro-prep ceramic hydroxylapatite column (20 μm, BIO-RAD laboratories, Hercules, CA, USA) that was previously equilibrated with 10 mM sodium phosphate at a pH of 6.8. Approximately 24-30 mg of seminal plasma total protein was loaded onto the column (1 cm × 10 cm) for each of 10 elution replicates (total of 240-300 mg total protein). The seminal plasma was allowed to bind to the hydroxylapatite slurry for 30 minutes. Elution was carried out at room temperature using a lineal gradient with 350 mM sodium phosphate, pH 6.8, and a flow rate of 0.5 ml/min. Two ml fractions were collected and their absorbance at 280 nm was measured. Fractions corresponding to each absorbance peak were pooled, concentrated and buffer-exchanged with phosphate buffered saline (PBS, pH 7.4) using a 20 ml ultra-filter device (Vivaspin, Sartorius, Goettingen, Germany) with a membrane cut-off of 5,000 Da. To examine protein band profiles, a sample of each fraction was reduced, denatured, and separated by electrophoresis on 12% polyacrylamide gel (SDS-PAGE) based on the protocol of Laemmli 1970. Gels were stained with Coomassie Blue R-250 (Sigma-Aldrich, St Louis, Missouri, USA) as described previously [19].

### Purification by fast protein liquid chromatography (FPLC)

Approximately 6-8 mg of a broad elution fraction from the hydroxylapatite column (Fraction C), corresponding to a prominent protein band at about 14 kDa, was loaded onto a gel filtration column (SEC, Hi Prep™ 26/60 Sepahacryl™ S-100, Amersham Laboratories, Piscataway, NJ, USA) in each of 10 elution replicates (60-80 mg total protein in Fraction C). The purification procedure was carried out at room temperature at a flow rate of 0.5 ml per minute using fast protein liquid chromatography (FPLC, Amersham Laboratories). Elution was performed isocratically using PBS at pH 7.4, and the absorbance of 4 ml fractions was measured at 280 nm. Fractions corresponding to each protein peak were pooled and concentrated in PBS at pH 7.4 using 20 ml ultra-filters with a membrane cut-off of 5,000 Da. Fractions were examined using SDS-PAGE, as described above.

### Bio-effects of purified protein fractions on ovarian function in llamas

The study was conducted from May to June using a herd of 55 non-lactating female llamas, ≥4 years of age and weighing 90-150 kg, at the University of Saskatchewan. A preliminary trial (n = 4 llamas per group) was done initially to test all fractions at the chosen dose. Llamas were given 5 mg Armour Standard LH (Lutropin-V, Bioniche Animal Health, Belleville, ON, Canada) i.m. to synchronize follicular wave emergence among animals [20]. Twelve days after LH treatment, llamas with a growing follicle ≥8 mm in diameter, determined by daily transrectal ultrasonography (7.5 MHz linear-array transducer, Aloka SSD 900, Tokyo, Japan), were assigned randomly to treatment groups and given 1.0 ml i.m. of a) PBS (negative control), b) whole seminal plasma (SP, positive control, 2.5 mg of total protein), c) Fraction A (1 mg), d) Fraction B (1 mg), e) Fraction C_1 _(1 mg), or f) Fraction C_2 _(1 mg). The ovaries were examined daily by transrectal ultrasonography from the day before treatment (Day -1) to Day 3 to detect ovulation, and every-other-day to Day 15 to monitor CL development, as described [5]. Ovulation was defined as the sudden disappearance of a large follicle (≥8 mm) that was detected during the previous examination, and was confirmed by later detection of a CL. The ensuing full experiment (n = 10 or 11 llamas per group) was similar to the preliminary trial except that Fraction C_1 _was omitted because it did not elicit ovulations in the preliminary trial and was not a prominent feature in the FPLC chromatogram or SDS-PAGE. Llamas used in the preliminary trial were also used in the full experiment by randomly assigning to a treatment group other than that assigned in the preliminary trial.

Blood samples for progesterone measurement were collected into heparinized tubes by jugular venipuncture on Day -1, 0, 1, and every-other-day to Day 15. Blood samples were centrifuged at 1700 × g for 25 minutes and the plasma was stored at -20°C. Plasma progesterone concentrations were determined using a commercial, double-antibody radioimmunoassay kit (Coat-a-Count total progesterone, Diagnostic Products Corporation, Los Angeles, CA, USA), as described previously [5,21]. All samples were analyzed in duplicate in a single assay. The intra-assay coefficients of variation were 4.5%, 5.1% and 3.6% for reference plasma progesterone concentrations of 1.7, 3.0, and 15.9 ng/ml, respectively.

Blood samples for LH measurement were collected from a subset of 5 randomly chosen llamas in each group. Blood was collected in heparinized tubes (5 ml) every 15 minutes for 8 hours starting immediately before treatment (Time 0 = treatment). A jugular catheter (inner and outer diameters of 1.0 and 1.5 mm, respectively) was fixed in place one day before treatment to minimize the effects of handling stress on plasma LH concentrations [5]. Blood samples were centrifuged at 1700 × g for 25 minutes and the plasma was stored at -20°C. Plasma LH concentrations were measured using a monoclonal LH antibody (518B7 lot #12) in a double-antibody radioimmunoassay [5,22]. Concentrations of LH are expressed in terms of NIAMDD-oLH-24. The minimum detectable limit of the assay was 0.13 ng/ml. The range of the standard curve was 0.06 ng/ml (93.8% ligand labeled LH) to 8 ng/ml (7.0% ligand labeled LH). The intra- and inter-assay coefficients of variation were 5.2% and 5.3%, respectively, for the high reference plasma LH concentration (1.15 ng/ml), and 9.7% and 10.2%, respectively, for the low reference plasma LH concentration (0.52 ng/ml).

### Statistical analyses

Data from the preliminary trial were analyzed separately from the main experiment and involved comparison of ovulation rate only. Single-point measurements (i.e., follicle diameter at the time of treatment, maximum CL diameter, day on which the CL was first detected, onset of CL regression) were compared between groups by analyses of variance. For serial observations (CL diameter, plasma LH and progesterone concentrations), data were centralized to the day of treatment (Day 0) and compared by analysis of variance for repeated measures using the Mixed Procedures of SAS (Statistical Analysis System Institute Inc., Cary, NC, USA) to determine the effects of treatment, time, and treatment-by-time interaction. When main effects or their interaction were significant (P ≤ 0.05), means at a given time were compared among groups by the method of least significant difference. Ovulation rates were compared among groups by Fishers exact test.

The experimental protocol was approved by the University Committee on Animal Care and Supply in accordance with the guidelines of the Canadian Council on Animal Care.

## Results

### Hydroxylapatite column and fast protein liquid chromatography

**Figure 1:**
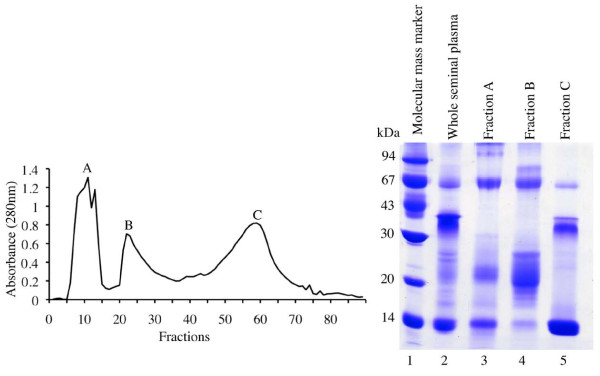
**Protein fractions of llama seminal plasma**. Fractions A, B and C were eluted on hydroxylapatite gravity chromatography columns using a lineal gradient of 10 to 400 mM sodium phosphate (left). Fraction C contained a major 14 kDa protein observed after denaturing on 12% SDS-PAGE (right).

**Figure 2:**
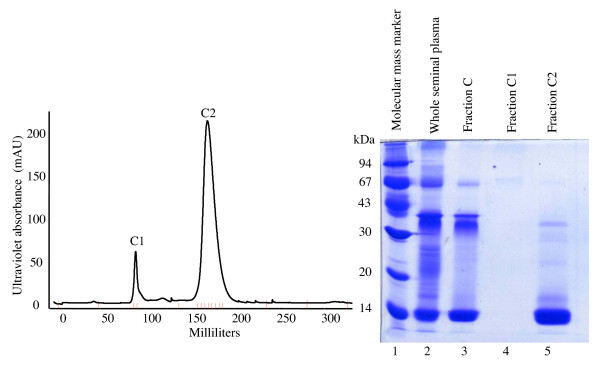
**Separation of protein Fraction C of llama seminal plasma**. Separation was done using sephacryl gel filtration fast protein liquid chromatography (FPLC) and isocratic elution with phosphate buffered saline (left). Fraction C was isolated previously by hydroxylapatite gravity column chromatography. Vertical lines along the x-axis represent fractions collected and examined. The protein band at about 14 kDa on denaturing 12% SDS-PAGE (right) was the major constituent of Fraction C_2 _(right).

Three protein fractions of llama seminal plasma (A, B and C) were eluted from a hydroxylapatite gravity chromatography column (Figure 1). Fraction C was comprised of a major 14 kDa protein, observed on denatured SDS-PAGE (Figure 1). Fraction C was subsequently loaded into a gel filtration column and separated into purified protein fractions using FPLC. Two sharp peaks (C_1 _and C_2_) were detected after gel filtration chromatography (Figure 2). The 14 kDa protein previously detected by hydroxylapatite column chromatography (Figure 1) was the major constituent of purified Fraction C_2 _identified by FPLC (Figure 2).

### Bioassay of purified protein fractions

In the preliminary trial, ovulations were detected only in groups treated with whole seminal plasma (positive control, 4/4) or Fraction C_2 _(4/4; Table 1). Based on this result, an expanded trial was done using a larger number of animals (Table 2). Ovulations were detected in >90% of llamas treated with either whole seminal plasma (positive control) or Fraction C2, and in few (Fraction B) or none (Fraction A and PBS) of the llamas in the other groups (Table 2). Subsequent to ovulation, the CL was detected earlier (P < 0.01) and attained a greater diameter (P < 0.01) in llamas treated with Fraction C_2 _than in those treated with whole seminal plasma (Table 3).

**Table 1:** Ovulation-inducing effect of protein fractions of llama seminal plasma (preliminary trial).

	PBS	Whole SP	Fraction A	Fraction B	Fraction C_1_	Fraction C_2_
Follicle diameter (mm) at treatment* (mean ± SEM)	9.7 ± 0.4	9.3 ± 0.6	11.0 ± 0.7	10.4 ± 0.5	9.6 ± 0.8	10.0 ± 0.7
Ovulation rate (%)	0/4^a^ (0%)	4/4^b^ (100%)	0/4^a^ (0%)	0/4^a^ (0%)	0/4^a^ (0%)	4/4^b^ (100%)

Female llamas (n = 4 per group) were given whole seminal plasma (SP, positive control), Fractions A or B (isolated by hydroxylapatite column chromatography), Fractions C_1 _or C_2 _(isolated by gel filtration chromatography), or phosphate buffered saline (PBS, negative control).

* No significant differences among groups.

^a,b ^Proportions with different superscripts are different (P < 0.03).

**Table 2:** Ovulation-inducing effect of protein fractions of llama seminal plasma (full experiment)

	PBS	SP	Fraction A	Fraction B	Fraction C_2_
Follicle diameter (mm) at treatment* (mean ± SEM)	8.1 ± 0.4	8.5 ± 0.4	8.8 ± 0.3	9.5 ± 0.6	8.9 ± 0.3
Ovulation rate (%)	0/10^a^ (0%)	10/11^b^ (91%)	0/10^a^ (0%)	2/11^a^ (18%)	10/11^b^ (91%)

Female llamas (n = 10-11 per group) were given whole seminal plasma (SP, positive control), Fractions A or B (isolated by hydroxylapatite column chromatography), Fraction C_2 _(isolated by gel filtration chromatography), or phosphate buffered saline (PBS, negative control).

* No significant difference among groups

^a,b ^Proportions with different superscripts are different (P < 0.001)

**Table 3:** Effect of protein fractions of llama seminal plasma on corpus luteum (CL) development in llamas (mean ± SEM)

	Seminal Plasma (n = 10)	Fraction B (n = 2)	Fraction C_2_ (n = 10)
Day CL detected (Day 0 = treatment)	2.9 ± 0.1^a^	2.5 ± 05^ab^	2.1 ± 0.2^b^
Maximum CL diameter (mm)	11.0 ± 0.4^a^	12.0 ± 1.0^ab^	13.3 ± 0.4^b^
CL diameter at Day 8 (mm)	10.4 ± 0.4^a^	11.3 ± 0.3^ab^	12.4 ± 0.4^b^
CL diameter on Day 15 (mm)	4.9 ± 0.2^a^	4.5 ± 0.5^a^	6.4 ± 0.5^b^

Female llamas were given whole seminal plasma (SP, positive control), Fraction B (isolated by hydroxylapatite column chromatography) or Fraction C_2 _(isolated by gel filtration chromatography).

^a,b ^Values with no common superscripts are different (P < 0.01).

**Figure 3:**
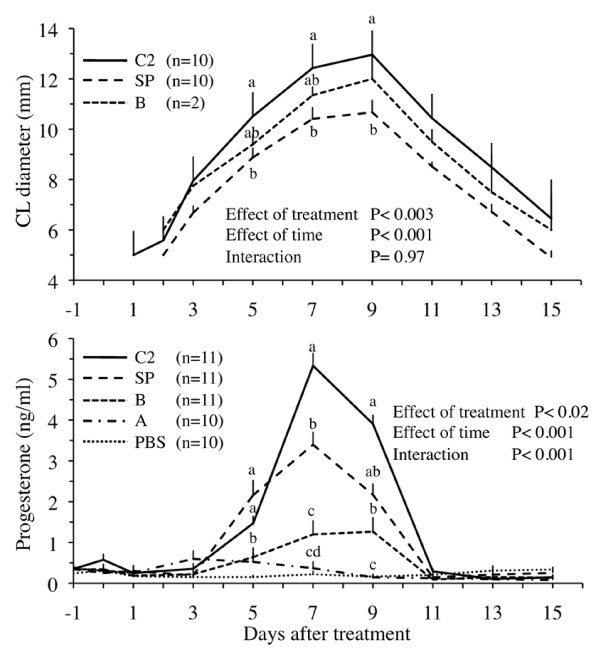
**Effect of protein fractions of llama seminal plasma on CL diameter and plasma progesterone concentrations in llamas**. Female llamas were given whole seminal plasma (SP, positive control), Fractions A or B (isolated by hydroxylapatite column chromatography), Fraction C_2 _(isolated by gel filtration chromatography), or phosphate buffered saline (PBS, negative control). ^abcd ^Within days, values with no common superscript are different (P < 0.05).

The day-to-day CL diameter profile was greater in llamas treated with Fraction C_2 _than in those treated with whole seminal plasma (Figure 3). Similarly, plasma progesterone concentrations were highest in llamas treated with Fraction C_2 _(Figure 3). Mean plasma progesterone concentration increased marginally in the Fraction B group as a result of only 2 ovulations. Progesterone concentrations remained basal in llamas treated with Fraction A or PBS.

**Figure 4:**
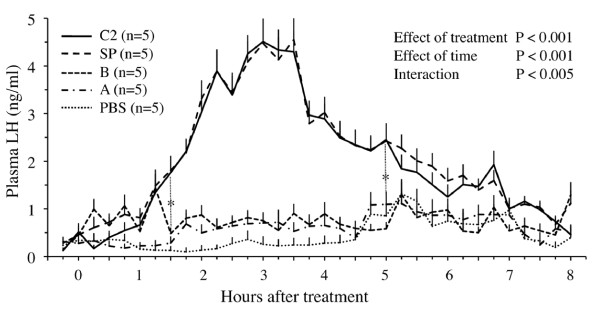
**Effect of protein fractions of llama seminal plasma on circulating LH concentration in llamas**. Female llamas were given whole seminal plasma (SP, positive control), Fractions A or B (isolated by hydroxylapatite column chromatography), Fraction C_2 _(isolated by gel filtration chromatography), or phosphate buffered saline (PBS, negative control). *Interval during which values in SP and C_2 _were higher (P < 0.05) than in other groups.

Plasma LH concentration surged during the 8-hour period after treatment (P < 0.01) in llamas treated with whole seminal plasma and those treated with Fraction C_2_, but remained unchanged in llamas treated with PBS or Fractions A or B (Figure 4). Plasma LH profiles were similar in llamas treated with whole plasma and those treated with Fraction C_2_; LH began to increase (P < 0.01) by 1.5 hours after treatment, peaked at 3 hours, and declined to pre-treatment levels by 7 hours after treatment.

## Discussion

A highly purified protein isolated from llama seminal plasma was identified as OIF using a combination of hydroxylapatite and gel-filtration chromatography. The purified protein elicited a preovulatory LH surge followed by ovulation and corpus luteum formation in llamas after intramuscular administration. The chromatographic strategy used in this study revealed a single bioactive fraction in llama seminal plasma with a molecular mass of approximately 14 kDa. In contrast, two bioactive fractions of Bactrian seminal plasma were detected in a study involving anion exchange chromatography, but molecular masses were not reported [14]. In this regard, the molecular mass of the protein isolated in the present study (based on the band pattern on denatured SDS PAGE) represents about half that found in a previous study [18] in which only the seminal plasma fraction greater than the nominal molecular mass cut-off 30 kDa (using centrifugal filtration devices) elicited ovulation in llamas. In the same study, however, partial enzymatic digestion with proteinase K did not ablate bioactivity despite rendering all proteins to ≤19 kDa [18]. Whether the 14 kDa protein identified in the present study is part of a larger protein complex or represents a bioactive pro-hormone form remains to be determined.

Results of the present and previous studies on seminal plasma are consistent with the hypothesis that OIF acts systemically to induce ovulation via a surge-release of LH, but results are not consistent with the hypothesis that OIF is related to GnRH. The molecular mass of the protein isolated in this study, and the effects of enzymatic digestion found in a previous study [18] document that OIF is a larger protein than GnRH, and the addition of anti-GnRH antibodies to in vitro culture of pituitary cells did not abolish the effect of seminal plasma on LH secretion [17]. The post-treatment surge in circulating concentrations of LH observed in the present study is consistent with that of previous studies in which intramuscular administration of llama or alpaca seminal plasma elicited an LH surge that began within 1 hour of treatment, peaked at 3 hours, and returned to baseline by 7 to 8 hours [5,9]. Whether OIF in seminal plasma mediates LH release solely at the level of the pituitary or by stimulating GnRH neurons in the hypothalamus remains unknown. However, LH release was more sustained in llamas given OIF than in those given GnRH [5], and the luteotrophic effects of OIF observed in the present study are consistent with the dose-dependent effects of seminal plasma reported in previous studies [5,9]. The more luteotrophic effect observed in llamas given purified OIF (Fraction C_2_) than in those given whole seminal plasma was unexpected and requires confirmation. It may be attributed to differences in absolute dose of OIF between the two treatments, though no differences in LH response were detected. Whether the difference in LH release between groups was too small to detect with our assay or the effect of OIF is also exerted at the local level (i.e., granulosa cells) remains to be elucidated.

An ovulation-inducing role of prostaglandins and estrogens in seminal plasma has been postulated in pigs [23,24], but results of the present and previous studies provide compelling documentation that OIF is neither a prostaglandin nor an estrogen [18]. The bioactivity of llama seminal plasma was not abolished by steroid extraction, and administration of fractions containing molecules <5 kDa did not induce ovulation [18]; prostaglandin and estrogen have molecular masses of 273 Da and 354 Da, respectively. Furthermore, elevations in circulating concentrations of estradiol during ovarian follicular wave development were not associated with LH release or ovulation in llamas and alpacas [25,26].

Results provide strong support for the hypothesis that ovulation in camelid species is elicited by an endocrine effect of OIF protein in seminal plasma. Camelids are classified as induced ovulators [27,28], but the results of early classic studies did not clearly distinguish between the effects of physical stimulation of copulation and exposure to constituents of the ejaculate. An important advantage over early studies is the availability of transrectal ultrasonography as a method to detect ovulation and CL development in more recent studies. Ultrasonographic detection of ovarian response in llamas and alpacas provides a rapid and robust in vivo bioassay for testing the effects of physical stimuli and different fractions of seminal plasma [5,7,18]. Collectively from 2 previous ultrasound studies [5,7], 42/45 (93%) llamas and alpacas ovulated after intramuscular administration of seminal plasma and 0/42 (0%) ovulated after transcervical intrauterine deposition of saline, with or without endometrial currettage. Similarly, the ovulation rate of llamas treated intramuscularly with whole seminal plasma or Fraction C_2 _in the present study was 93%. Using the same llama ovulation bioassay, OIF has also been detected in the seminal plasma of other species (bull, stallion, boar, rabbit), suggesting that it is widely conserved [8,10,29].

Identification of the amino acid sequence and structural form of the OIF protein will be important in developing tools to examine the mechanism of action of OIF, including the tissue source within the male and the tissue targets within the female. Development of tools to measure OIF and OIF receptors will also permit test of the hypothesis that some as yet unexplained causes of infertility are based on alterations in the sensitivity to, or abundance of, this molecule. Recent documentation of the presence of OIF in the seminal plasma of several mammalian species suggests an evolutionary link between species classified as induced or spontaneous ovulators. Further characterization of OIF is needed to determine the relative prevalence and functional role of OIF among species.

In conclusion, OIF was isolated from llama seminal plasma as a 14 kDa protein molecule using a two step combination of hydroxylapatite and gel filtration chromatography. Systemic administration of purified OIF resulted in a preovulatory LH surge followed by ovulation and CL formation in llamas suggesting an endocrine effect at the level of the hypothalamus (release of GnRH) or the pituitary (gonadotrophs).

## Competing interests

The authors declare that they have no competing interests.

## Authors' contributions

MR participated in designing the study, acquisition, analysis and interpretation of data, and in writing and revising the manuscript. LTJD, YAL and RAP participated in the acquisition and interpretation of the data. As Principal Investigator, GA participated in the intellectual and experimental design of the study, the acquisition, analysis and interpretation of data, as well as writing and revising the manuscript. All authors read and approved the final manuscript.
